# Himalayan watersheds in Nepal record high soil erosion rates estimated using the RUSLE model and experimental erosion plots

**DOI:** 10.1016/j.heliyon.2023.e15800

**Published:** 2023-05-02

**Authors:** Prayon Joshi, Raize Adhikari, Rajendra Bhandari, Bibek Shrestha, Nischal Shrestha, Samikshya Chhetri, Subodh Sharma, Joyanto Routh

**Affiliations:** a Aquatic Ecology Centre, Kathmandu University, Nepal; b Department of Environmental Science and Engineering, Kathmandu University, Nepal; c Department of Thematic Studies - Environmental Change, Linköping University, Sweden

**Keywords:** Empirical models, Agriculture, Nepal mid-hills, Runoff plots, Soil erosion

## Abstract

The rising unpredictability in the food supply chain in many parts of the world is related to soil loss and poor agricultural output. The Revised Universal Soil Loss Equation (RUSLE), widely used for estimating soil loss, was applied in the western mid-hills in Nepal, with steep slopes and fragile geology. This region is at high risk for rapid soil erosion and mass wasting. To estimate soil loss, this study utilized the RUSLE model with experimental erosion plots in the Aadhikhola and Tinahukhola watersheds, capturing real-time erosion in the field. The annual soil loss for the Aadhikhola watershed is estimated at ∼41.4 tons ha^−1^ yr^−1^. In contrast, in the Tinahukhola watershed, soil loss is low (∼24.1 tons ha^−1^ yr^−1^). Although annual rainfall showed an increasing trend in both watersheds, the change in soil loss was statistically insignificant. The high erosion rates from the experimental plots in both watersheds support the model outputs. Results from the experimental plots recorded the rate of soil erosion for different land use as: irrigated agricultural land > rainfed agricultural land > forests. The trends highlight the role of human activities in enhancing soil erosion in these mountainous terrains in terms of medium to long-term perspectives. Therefore, sustainable agriculture practices in these terrains must investigate alternate ways to decrease soil erosion to support people's livelihoods.

## 1 Introduction

Precipitation is the most prominent agent fostering soil erosion along young and fragile mountain landscapes dominated by monsoons [1]. Such episodic monsoon showers affect soil productivity and agricultural output. For example, 9% of crop productivity has declined in Africa [2]. Similarly, in Europe, the erosion rate was ∼2.46 tons ha^−1^, 1.6 times more than the rate of soil formation [3]. In Asia, ca. 663 million hectares of land are affected by soil erosion, which is ca. 20 tons ha^−1^ yr^−1^. The main hotspots are China, India, and Indonesia. In the last 20–100 years, the rapid increase in human population and agriculture has worsened the situation in countries like China, Nepal, Lebanon, etc. [4].

Previous studies indicate that factors like rainfall and surface runoff, agricultural activities, vegetation cover, and slope result in differential soil loss patterns [5]. In addition, rapid growth in the human population and non-sustainable land-use practices enhance soil loss in the watersheds. Reports suggest that one-third of the world's total arable land has been lost due to soil erosion in the last 40 years, and it continues at a rate of 10 million hectares annually [6,7]. In particular, converting natural forests to agricultural croplands cause severe negative impacts leading to high soil erosion due to reduced vegetation cover [8,9]. These assessments predict that the world's topsoil could become unproductive within 60 years [9], which has grave consequences.

Nepal is facing unregulated and haphazard construction and land-use changes in the hills, making them more vulnerable to erosion and landslides; it destabilizes the fragile geology and alterations in local hydrological conditions [10]. Moreover, intense rainfall, mainly from June–September, has made the steep slopes vulnerable to high soil erosion and collapse [11,12]. According to the Ministry of Forests and Environment report [12], the average rainfall is projected to increase from 2.1 to 4.5% by 2045 and increase to 10.7–23% by 2100. The increasing precipitation trend, coupled with conventional tilling practice in mountainous watersheds, can quickly sweep away topsoil from the steep slopes [13]. This makes the fragile slopes of the Nepal mid-hills highly vulnerable to soil erosion and substantial loss.

Agricultural land in Nepal constitutes 28% of land cover of which 21% is cultivated, and 7% remains uncultivated [14]. Land degradation, mainly soil erosion affects agriculture and is a major challenge in Nepal [13]. Conventional tillage practice, observed in arable lands in Nepal, leads to significant loss of topsoil during the rainy season due to steep and fragile landscapes situated on sloping hills [15]. Consequently, the amount of soil loss from conventional tillage is 5.5 Mg ha^−1^ (1 Mg = 1000 kg) more than the reduced tillage system, and it is accompanied by a decline in nutrients due to enhanced soil loss [15]. Previous studies indicate that the degradation of arable land in Nepal includes the structural deterioration in soil caused by the increase in bulk density and runoff, and a decrease in porosity [13]. The use of tractors and harvesters for tillage results in soil compaction. Similarly, pastures are used for free grazing of animals, resulting in enhanced soil compaction and removal of vegetation and leaf litter. As a result, this soil is more susceptible to mass wasting and soil loss. In 2006, it was reported that 0.2 million ha of land was affected by soil degradation, accounting for 1.4% of arable land in Nepal [13]. Poor land management led to a decline in soil quality and crop production, and realistic plans are urgently needed to enhance local participation to decrease land degradation [14].

The Universal Soil Loss Equation (USLE), developed by Wischemeir and Smith in 1965 and further improved in 1978, is the most widely used empirical model [16]. RUSLE accounts for erosion due to rainfall and comprises five main factors, which are rainfall erosivity (R), soil erodibility (K), slope length (L), steepness (S), cover factor (C), and support practice factor (P) [16]. This model has been frequently used to estimate soil loss in small watersheds, including those in Nepal [17]. Although empirical models like RUSLE are simple and helpful in assessing soil erosion, these models require additional practical/on-site verification processes for broader applicability and better predictability [18]. One of the significant limitations of this model is the precise estimation of deep-seated gully erosion. As a result, erosion rates predicted by RUSLE have a low threshold in watersheds exposed to gully and large-scale mass movement, e.g., the active occurrence of landslides. Hence, runoff plots for erosion have been used widely for a better assessment of topsoil loss and management practices and to verify model outputs [19,20]. Several parameters are weighed carefully while constructing these plots, e.g., precipitation, amount of soil transported, surface evaporation, runoff, infiltration, moisture, fertility, and agricultural output, providing a robust assessment [19]. Most existing studies on soil erosion consider the average precipitation values derived from sensors for modeling. The main drawback is that sensors lack the flexibility to mimic the dynamism of natural rainfall [21]. Similarly, laboratory studies cannot fully simulate the complicated interaction of precipitation with surface features in any watershed.

The mid-hills in Nepal are prone to extensive soil erosion [22,23]. However, regional studies on soil erosion have used models without any field plot methods or corroboration of the proposed inferences [22] based on models. Utilizing both schemes involving soil erosion models and erosion plots for estimating soil loss at the watershed level is a more robust and accurate practice for long-term predictions and implementing soil conservation measures at the watershed level [15]. Therefore, in this study, we aim to estimate the soil erosion rate using empirical models in conjunction with field experiments in watersheds from the mid-hills of Nepal. We observed moisture, temperature, and electrical conductivity with the help of soil sensors installed 15 cm below the ground level and taking continuous measurements of various physical parameters in both watersheds. In addition, we examined the effect of precipitation on erosion under different land use practices in these mountainous terrains. A better understanding of erosion in these watersheds has broad interests due to its influence on diverse geological landscapes, soil types, vegetation, and climate change effects within a narrow geographic terrain in the Himalayan mid-hills sensitive to natural and anthropogenic changes [20]. In particular, the modeling output combined with the field experimental data from these mountainous terrains could help better assessment of sudden or incremental precipitation and land-use practices in these catchments.

## 2 Methods

### 2.1 Study area

**Fig. 1:**
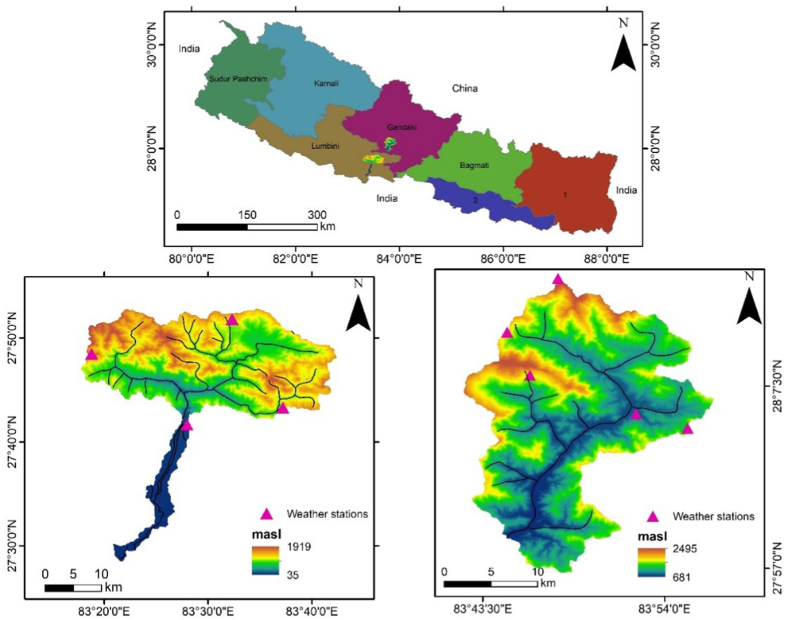
Tinahukhola and Aadhikhola watersheds are shown in the provincial map of Nepal (elevation layer extracted from ASTER DEM, Weather stations from DHM Nepal).

We selected two watersheds, Tinahukhola and Aadhikhola (Fig. 1), representing the typical mid-hill terrains in Nepal. Tinahukhola watershed stretches along the Rupandehi and Palpa districts in Lumbini province, with an area of 1081 km^2^ [24]. The altitudinal range varies from 85 to 1940 m above sea level. The watershed is drained by the Tinahukhola River, which is a monsoon-dominated perennial river originating from the southern slopes of the Mahabharata Range in the Palpa district [25]. The significant elevation difference has resulted in diverse climatic conditions ranging from sub-tropical warm to humid conditions. The average annual rainfall ranges from 1500 mm on the northern side and 2400 mm on the southern side of the watershed [26]. The major soil types within this watershed are fertile eutrophic cambisols and fluvisols [27]. Geologically, this watershed is mainly dominated by sedimentary and metasedimentary rocks upstream, while thick sandstone beds (1–45 m) are observed in the mid-section. The downstream Terai plains have rich alluvial sediments containing boulders, gravel, silt, and clay [28]. The hilly region of the Tinahukhola watershed is dominated by forests (62%), followed by agricultural land (33%); the floodplains in the Terai region have urban settlements with patches of croplands [20].

On the northern side of Tinahukhola lies the Aadhikhola watershed, with an area of 195 km^2^ [24], located in the Syangja district of the Gandaki province. The rainfall in this region ranges from 2800 to 3200 mm; monsoons contribute around 80% of the precipitation [29]. Aadhikhola watershed includes soil types dominated by fluvisols, cambisols, and minor gleyic luvisols [27]. The temperature ranges from 5 to 32 °C [29]. This watershed lies in the Lesser Himalayan Zone in Nepal. The geology is dominated by non-fossiliferous, sedimentary, and meta-sedimentary rocks such as slate, phyllite, schist, quartzite, limestone, and dolomite, ranging from Precambrian to Eocene ages [28]. Like the Tinahukhola watershed, the Aadhikhola watershed has a dominant forest cover. This is followed by shrubland and cropland, covering nearly 27% of the area, whereas the built-up area, grassland, and water bodies cover only 7% of the total area [30].

### 2.2 Estimation of soil loss from RUSLE

**Fig. 2:**
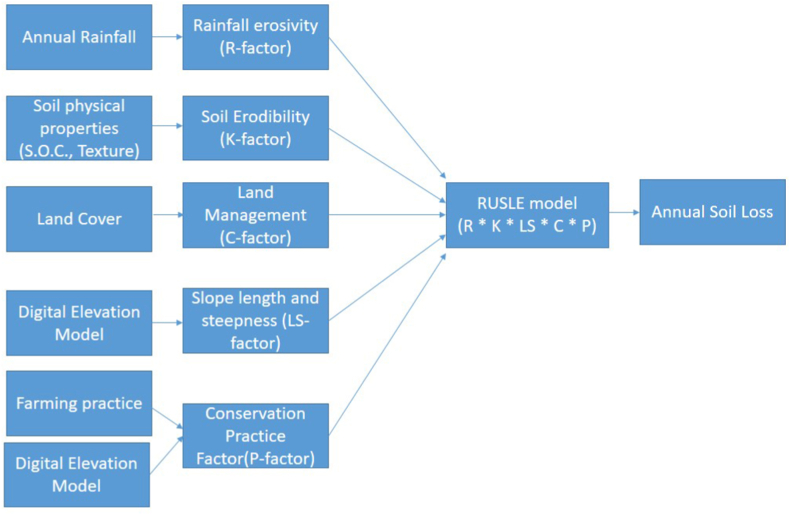
A methodological framework for the implementation of the RUSLE model in our study.

The RUSLE model (Equation I) was used to determine the annual soil loss induced by slope runoff, specific cropping, and management practices [31]. The schematic framework of this assessment is shown in Fig. 2.

(1) A=[R]*[K]*[LS]*[C]*[P]

where, A denotes the annual soil loss, and provides value for the spatial and temporal average soil loss (tons ha^−1^ yr^−1^), R (MJ mm ha^−1^ h^−1^ yr^−1^) is the rainfall-runoff erosivity factor, K (tons h MJ^−1^ mm^−1^) is the soil erodibility factor, LS (unitless) indicates slope length, gradient, C (unitless) represents land cover, and P (unitless) is conservation practices.

#### 2.2.1 Rainfall erosivity factor (R-factor)

Rainfall erosivity represents the effect of rainfall volume and intensity as a main driving factor for water-induced soil loss. The rain erosivity factor was obtained based on the precipitation data from the Department of Hydrology and Metrology (DHM) using equation II [23]:

(2) fall[R]=38.5+0.35*[P]

where P = mean annual precipitation.

This data was extrapolated to the whole watershed using the inverse distance weightage method [32].

#### 2.2.2 Soil erodibility factor (K-factor)

The soil erodibility factor measures the susceptibility of the soil particles to detach and transport due to rainfall and runoff [22]. The erosion rate in a standard plot with a slope length of 22.13 m and a 9% slope gradient is the slope erodibility factor reflecting the soil loss based on the rainfall erosivity index [33]. In our study, we used the empirical relationship with soil properties from different soil samples collected from watersheds to calculate the soil erodibility factor [33]. The textural composition was obtained using the Bouyoucos hydrometer method [34]; soil organic matter was obtained using the dry combustion method [35]. Then, following the methods described earlier [36], the soil erodibility factor was obtained using Equation (3).

(3) $$K=F_{csand}*F_{si-cl}*F_{orgc}*F_{hisand}*0.1317$$

which can further be divided into the following equations:

3(a) $$F_{csand}=\left[0.2+0.3\;\exp\left\{-0.0256\left(1-\frac{SIL}{100}\right)\right\}\right]$$

3(b) $$F_{si-cl}={\left(\frac{SIL}{CLA+SIL}\right)}^{0.3}$$

3(c) $$F_{orgc}=\left(1.0-\frac{0.25C}{C+\exp\left(3.72-2.95C\right)}\right)$$

3(d) $$F_{hisand}=\left(1.0-\frac{0.70SN1}{SN1+\exp\left(-5.51+22.9SN1\right)}\right)$$

where, SAN= Sand percentage, SIL= Silt percentage, CLA = percentage of Clay, C = Soil Organic Carbon, SN1=Sand content subtracted from 1 divided by 100.

The data obtained were extrapolated to the whole watershed using the Inverse Distance Weighing technique [[37], [38],^39^].

#### 2.2.3 Slope length and steepness factor (LS – factor)

The digital elevation map was obtained from the ASTER platform. The spatial resolution of this image was 15 m, which was converted to 30 m resolution to match the cell size with the LANDSAT image (used for C-factor). The slope length denoted by L (equation IV) and slope gradient represented by S (equation V) were obtained previously for the mid-hills of Nepal [40]. The slope length and steepness map were obtained based on previous literature [41].

(4) $$L={\left[\frac{cell\;size}{22.13}\right]}^{m}$$

where m = 0.2 for slope <1%, m = 0.3 for slope = 1–3%, m = 0.4 for slope = 3–4.5% and m = 0.5 for slopes >4.5, L = slope length factor, Cell size = cell size of the DEM map used

(5) $$S=0.0138+0.0097*s+0.00138*s^{2}$$

where S = slope steepness factor, *s* = slope in percentage.

#### 2.2.4 Land management factor (C-factor)

LANDSAT images (<10% cloud cover) from November 2018 were processed. While using the satellite images, we opted for less cloud cover (<10%) and synchronized other relevant data (e.g., rainfall, soil properties), which are used for calculating the RUSLE factors. Various corrections were included to account for atmospheric conditions, radiometric calibration, and geometric correction. The images were classified with the maximum likelihood algorithm. Various land-use classes were assigned to different C-factor values provided earlier and shown in Table 1 [3].

**Table 1:** Details of factor values for management cover and support practice factor.

C-factor values based on different land use (Panagos et al., 2015)		P-factor values for contour practice based on slopes (Shin, 1999)	
**Land Use**	C-Factor	Slope percentage	P-Factor
**Forest**	0.03	0–7	0.55
**Agricultural**	0.21	7–11.3	0.6
**Barren**	0.45	11.3–17.6	0.8
**Water Body**	0	17.6–26.8	0.95
**Built-Up**	0	>26.8	1

#### 2.2.5 Conservation practice factor (P-factor)

The support practice factor provides the rate of soil erosion based on various cultivated practices in the study region. The value of the P-factor depends on the type of cropping practices done in the watershed, whether it is contouring, terracing, strip cropping, etc. Based on the land use practices, the slope values and the P-factor is assigned, ranging from 0 to 1, 0 being the least soil erosion and 1 being the highest rate of soil erosion [42]. In our study area, we mainly observed the practice of contouring. Thus, respective values were assigned as given in Table 1.

### 2.3 Observation of response of soil erosion to the change in rainfall

Based on the annual precipitation obtained from various sites within the study area, the average rainfall for five years was calculated from 1978 to 2017. The annual precipitation was used to calculate erosivity due to rainfall (R-factor) for the specific time interval. The R-factor was used in the RUSLE equation keeping the other factors (K-factor, LS-factor, C-factor, and P-factor) constant (same as in 2018), which yielded a change in soil loss due to variation in rainfall.

### 2.4 Experimental plots

We established experimental plots in three land-use settings (forests, rain-fed agricultural land, and irrigated agricultural land) to assess the impacts of rainfall on topsoil erosion (Table 2). Irrigated agricultural land is intensively utilized (3–4 crops a year) compared to rainfed agricultural land (1–2 crops a year). Therefore, data obtained from these plots are beneficial for comparing the erosion rates [19,43] calculated from the RUSLE model. Three replicate plots of dimension 6 × 3 m^2^ were constructed in each land-use setting in both watersheds [44]. Manual funnel-type rain gauges were placed on each plot to measure rainfall. In addition, a gutter was placed, so the overland flow from the plots was collected into metallic drums to track the net discharge.

**Table 2:** Description of plots that were used in this study.

**Plot name**	Watershed	Land Type	Locations	**Elevation (masl)**
**AB**	Aadhikhola	Rainfed Agricultural Land	28° 6′10.53″N	881
			83°53′32.14″E	
**AK**	Aadhikhola	Irrigated Agricultural Land	28° 6′7.33″N	862
			83°53′26.90″E	
**AF**	Aadhikhola	Forest	28° 6′13.19″N	896
			83°53′31.81″E	
**PB**	Tinahukhola	Rainfed Agricultural Land	27°51′27.36″N	1054
			83°32′44.33″E	
**PK**	Tinahukhola	Irrigated Agricultural Land	27°51′25.86″N	1039
			83°32′48.18″E	
**PF**	Tinahukhola	Forest	27°51′27.70″N	1064
			83°32′42.81″E	

The data collection was done for the 2019 monsoon season. The measurements included precipitation amount and the overflow into drums after each rainfall event. The sample taken from the drum was transported to the laboratory to determine suspended solids. The relation between rainfall and the amount of soil transported during each rainfall event was selected from the specific land-use class for both watersheds. Further soil samples from each replicate and land-use plots from both watersheds were collected for bulk density estimation. The samples were collected at two depths (0–15 cm and 15–30 cm) to include top and subsoil profiles. Studies have been published to document the variability in topsoil erosion due to the differences in soil bulk densities [45]. In this study, we focused on rainfall and soil loss during the peak monsoons (∼2 months), which coincides with the major duration for topsoil erosion in the mid-hills of Nepal.

#### 2.4.1 Precipitation, overland flow, and soil loss

The funnel rain gauge measured the precipitation in the experimental plots. The manual readings were further verified with data obtained from DHM, Nepal. Precipitation contributes to subsurface flow (percolation and infiltration) and overland flow. The overland flow is responsible for soil transport; hence tanks were used to collect the overland flow. The water height in the tanks was used to estimate the overland flow volume.

$$Volume=\pi *\frac{square\;of\;tank\;diameter}{4}\;*hieght\;of\;tank$$

Additionally, samples from the tanks were taken to estimate the total suspended load. Finally, the amount of sediment transported (lost) during each rainfall event was calculated from the suspended load and the overland flow volume.

Totalsoilloss=suspendedload*volumeofoverlandflow

After each sampling event, the drums were emptied and cleaned. All the drums, after cleaning, were ready to collect fresh runoff water from the plots during rainfall.

#### 2.4.2 Soil water content, conductivity, and temperature

We installed EM-50 sensors in locations representing major land-use classes in the Tinahukhola watershed. These sensors were installed following guidelines provided by the manufacturer for recording soil moisture (cm^3^ cm^−3^), electrical conductivity (mS cm^−1^), and temperature (^⁰^C). These sensors use the dielectric method for measuring moisture, a thermistor for temperature, and an electromagnetic field for electrical conductivity. The sensors were programmed to take measurements every 6 h during the monsoons (see Supplementary data; Table 4). In addition, three sensors were placed in the selected land-use settings for automated measurements.

#### 2.4.3 Revised universal soil loss equation (RUSLE) at plot level

To validate the RUSLE model output in these watersheds, we compared this with data generated from the experimental plots. The precipitation value obtained from the rain gauges installed within plots was used for the R-factor. Similarly, for the inputs of the K-factor, soil samples were collected from the plots and analyzed in the lab; the values were used in equation II. The slope length factor (L) for these plots was 6, whereas the slope steepness was measured with a clinometer, and the value was used in equation V for calculating the slope steepness factor (S). For agricultural land, irrigated and rainfed plots were assigned a value of 0.21 for the C-factor, whereas the forest was assigned a value of 0.03 [3]. The P-factor was based on the slope percentage calculated from the clinometer [42].

## 3 Results

### 3.1 Model parameters

The R factor determines the soil erosion loss due to rainfall [46]. The average rainfall erosivity of the Aadhikhola watershed was estimated to be 1071 MJ mm ha^−1^ h^−1^ yr^−1^ (Supplementary data; Fig. 1). The erosion rate is higher than the rainfall erosivity factor in the Tinahukhola watershed and is estimated as 733 MJ mm ha^−1^ h^−1^ yr^−1^. The K factor measures the vulnerability of soil particles to be eroded by rainfall. It depends upon soil properties like soil texture, organic matter present, soil structure, and permeability [47]. The soil erodibility factor in both watersheds is similar. It is 0.092 tons ha MJ^−1^ mm^−1^ in Aadhikhola and 0.096 tons ha MJ^−1^ mm^−1^ in Tinahukhola (Supplementary data; Fig. 2).

**Fig. 3:**
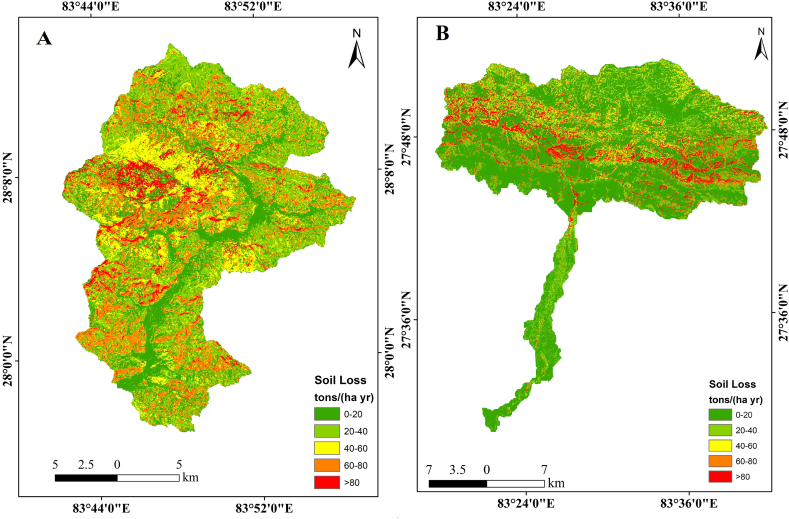
Map showing soil erosion rate in watersheds: A – Aadhikhola, B – Tinahukhola.

The topographic feature of the watershed is taken as the LS factor in the model. An increase in this factor denotes steepness and longer slope length, making the soil more prone to erosion. The long and steep slopes of the Aadhikhola watershed contribute to the high LS factor of 4.36, whereas it is 1.81 in Tinahukhola (Supplementary data; Fig. 3).

**Fig. 4:**
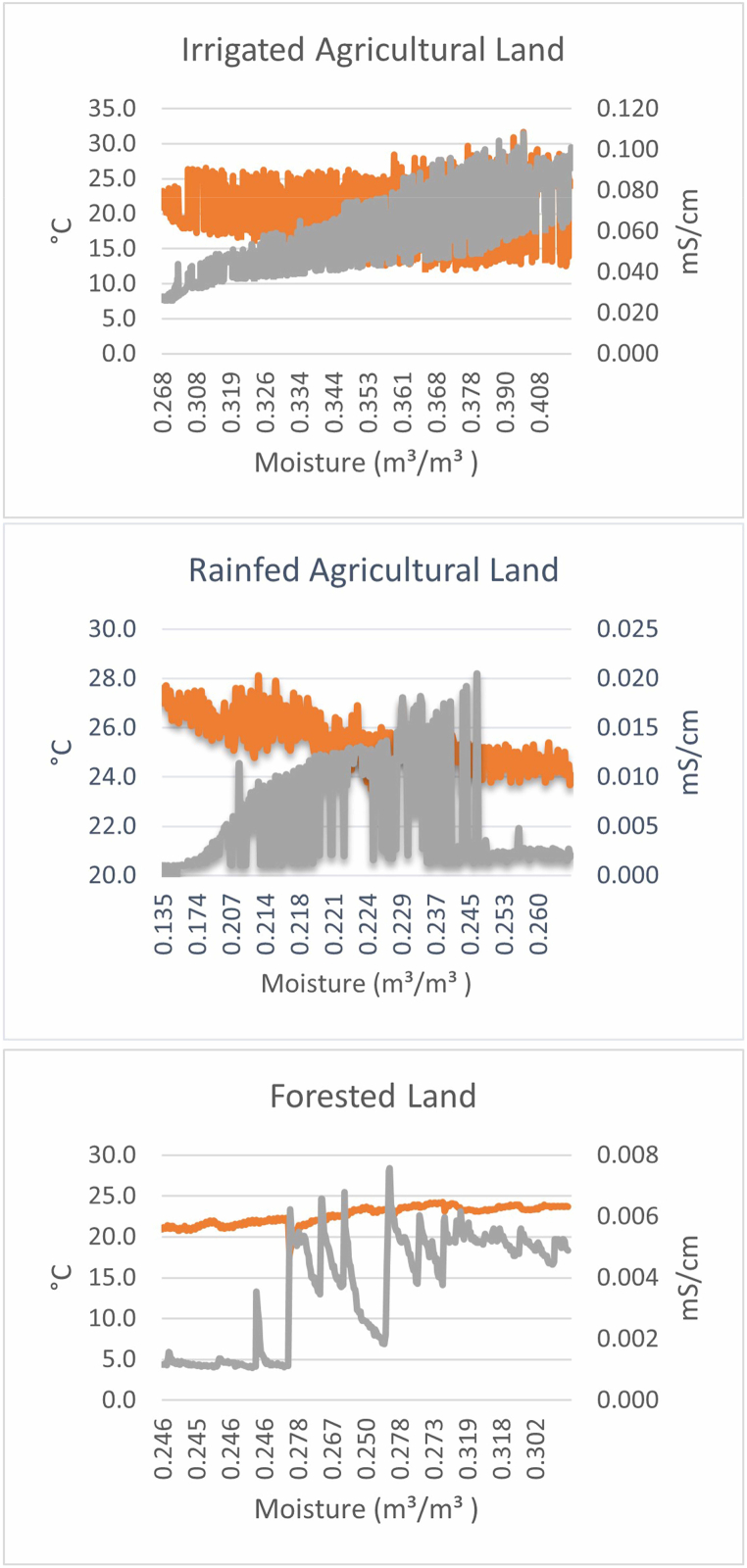
Relationship of moisture content with temperature and electrical conductivity in experimental plots of Rainfed cropland, Irrigated cropland, and Forested area in the Tinahukhola watershed.

The land cover in any area is vital in affecting soil erosion. The site with relatively high vegetation cover has a low rate of soil erosion. In contrast, the area with less vegetation has a high rate of soil erosion. The C-factor is determined based on the land-use classification. The overall accuracy was 86.1% and 87.4%, with Kappa coefficients of 0.82 and 0.84 for the Aadhikhola and Tinahukhola watersheds, respectively (Supplementary data; Table 1, Table 2). The classification scheme is very reliable, with an overall accuracy of >80% and a Kappa coefficient of >0.80 [48]. Various C-factor values are assigned to different land-use classes, e.g., barren land exposed to high erosion is assigned a high C-factor value. In contrast, waterbody and built-up areas are assigned a value of 0. The C-factor in the Aadhikhola watershed was 0.11, whereas it was 0.13 for the Tinahukhola watershed (Supplementary data; Fig. 4).

**Fig. 5:**
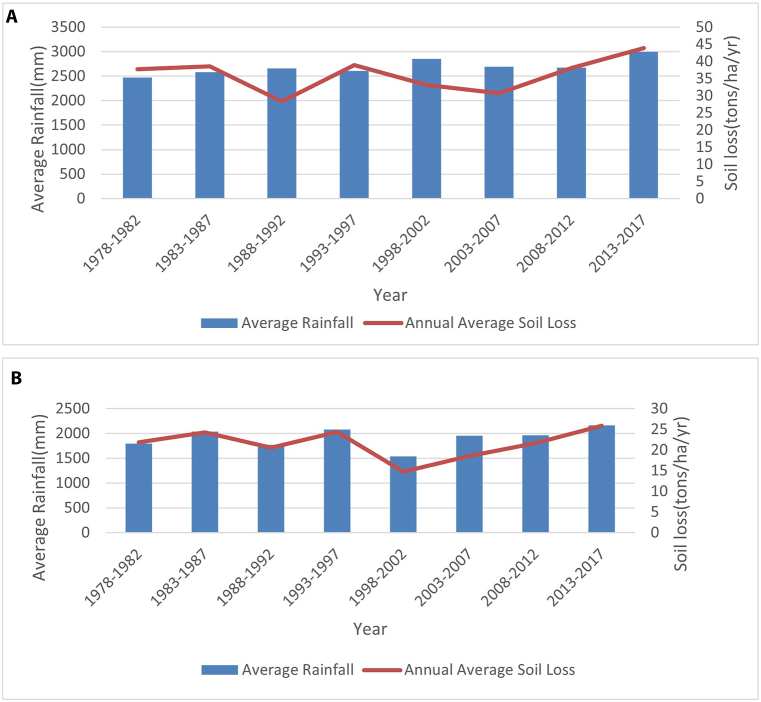
Graph showing the long-term relationship between soil loss and precipitation: A – Aadhikhola watershed, B – Tinahukhola watershed.

The P-factor or the conservation practice factor refers to techniques practiced reducing soil erosion. For example, contour farming, strip cropping, and terracing are practices that affect soil erosion. The P-factor is similar in both watersheds, i.e., 0.95 in Aadhikhola and 0.93 in Tinahukhola (Supplementary data; Fig. 5). The P-factors were almost identical as both watersheds practice contour farming as protective measures.

### 3.2 Annual rate of soil loss

We combined all the factors incorporated in the RUSLE equation and calculated the soil erosion loss from both watersheds. Based on this, soil loss from the Aadhikhola watershed is 41.4 tons ha^−1^ yr^−1^ with a maximum value of 1302 tons ha^−1^ yr^−1^ and a minimum of 0.09 tons ha^−1^ yr^−1^. The loss mainly occurred in the mid-western part of the watershed (Fig. 3). Similarly, soil loss from the Tinahukhola watershed is estimated as 24.1 tons ha^−1^ yr^−1^ with a maximum of 278 tons ha^−1^ yr^−1^ and a minimum of 0.04 tons ha^−1^ yr^−1^. Again, the loss is mainly from the mid-northern part of the watershed (Fig. 3).

### 3.3 Precipitation amounts and soil loss in experimental plots

The relation between the amount of rainfall that occurred and soil loss in different land-use classes is summarized in Table 3. We captured 15 rainfall events (observations) in the Tinahukhola watershed during the 2019 monsoon season. The mean amount of precipitation recorded in three replicates of the irrigated agricultural plot is 867 ± 4.87 mm. The amount is comparable to the rainfed agricultural plot with a value of 898 ± 15.4 mm. However, the plots in forests recorded a lower amount (688 ± 1.74 mm). In the Aadhikhola watershed, 13 rainfall events were recorded. Like the Tinahukhola watershed, the plots in forests record less precipitation (450 ± 26.0 mm) compared to irrigated agricultural plot (458 ± 0.84 mm) and rainfed-agricultural plot (475 ± 8.02 mm).

**Table 3:** Bulk density of soil and soil loss due to precipitation.

Watershed	Land use	Observed rainfall events	Precipitation (mm)	Sediment Load (tons/ha)	Bulk density (gm cm^−3^)	
					0–15 cm	15–30 cm
Tinahukhola	Irrigated agricultural land	15	867 ± 4.87	25.7 ± 0.16	1.10 ± 0.13	1.38 ± 0.23
	Rain-fed agricultural land	15	898 ± 15.4	19.4 ± 0.01	1.23 ± 0.12	1.57 ± 0.19
	Forests	15	688 ± 1.74	14.3 ± 0.13	1.17 ± 0.02	1.28 ± 0.09
Aadhikhola	Irrigated agricultural land	13	458 ± 0.84	26.9 ± 0.47	1.48 ± 0.11	1.33 ± 0.12
	Rain-fed agricultural land	13	475 ± 8.02	15.2 ± 0.19	1.30 ± 0.10	1.42 ± 0.04
	Forests	13	450 ± 26.0	6.47 ± 0.11	1.55 ± 0.07	1.63 ± 0.02

The topsoil erosion, and the overland flow in experimental plots, show the trend: Irrigated agricultural land > Rainfed - agricultural land > Forests (Table 3) in both watersheds. In the Tinahukhola watershed, the amount of erosion in the irrigated agricultural plot is 25.8 ± 0.16 tons ha^−1^. Similarly, the amount in the rainfed agricultural and forests is 19.4 ± 0.01 tons ha^−1^ and 14.3 ± 0.13 tons ha^−1^, respectively. The variability in soil erosion in the Aadhikhola and Tinahukhola watersheds is similar.

The plots in the irrigated agricultural land record the highest erosion with 26.9 ± 0.47 tons ha^−1^, followed by rainfed agricultural plots (15.2 ± 0.19 tons ha^−1^). The forests record the lowest erosion with a value of 6.47 ± 0.11 tons ha^−1^. While the total soil loss observed is higher in Tinahukhola for the rain-fed agricultural plot and forests, the total soil loss in the irrigated agricultural plots at Aadhikhola is more than in Tinahukhola. This higher soil loss observed in Aadhikhola than in Tinahukhola for irrigated land might be due to the differences in agricultural practices between the two sites. Intense tilling and the absence of crop cover may influence soil loss in the watershed.

The RUSLE model when applied to the plot level showed a similar result to that of the observed value except for the irrigated agricultural land (Table 5). From our observation, we reported that for the Tinahukhola watershed, the value from the RUSLE model was slightly higher for the rainfed agricultural land compared to the observed value (difference = 2.31 tons ha^−1^). Similarly, the difference of 4.854 tons ha^−1^ was seen in the Aadhikhola watershed. On the other hand, the difference between the model and observed values is even smaller for the forested land use type. For the Tinahukhola watershed, the observed value was slightly higher than the model estimates (1.78 tons ha^−1^). In comparison, for the Aadhikhola watershed, the observed value was slightly higher than the model estimates (0.344 tons ha^−1^). A significant difference between the model estimation and the observed values were observed for the irrigated agricultural land use type in both watersheds. For the Tinahukhola watershed, the observed value is 11.12 tons ha^−1^ more than the estimation of the model, while in the Aadhikhola watershed, it is 17.74 tons ha^−1^ high.

### 3.4 Soil bulk density and EM-50 sensor records in plots

The results for soil temperature, moisture, and electrical conductivity from the EM-50 sensors at the Tinahukhola watershed are summarized in Table 4. Similarly, bulk densities at two different depths (0–15 cm and 15–30 cm) for both watersheds are summarized in Table 3. The temperature in the experimental plots at Tinahukhola is rainfed agricultural plot (25.48 °C) > forests (22.65 °C) > irrigated agricultural plot (20.55 °C). The moisture content is higher in the irrigated agricultural land (0.35 m^3^ m^−3^), followed by the forests (0.27 m^3^ m^−3^) and rainfed agricultural land (0.22 m^3^ m^−3^). While the electrical conductivity is high in the irrigated agricultural land (0.06 mS cm^−1^), it is comparable to the rainfed agricultural plot (0.01 mS cm^−1^) and forests (<0.01 mS cm^−1^).

**Table 4:** Minimum, maximum, and average values of temperature, moisture, and electrical conductivity from EM-50 sensors.

Land use type	Temperature (^o^C)			Moisture (m^3^/m^3^)			Conductivity (mS/cm)		
	Min	Max	Average	Min	Max	Average	Min	Max	Average
Irrigated Agricultural Land	26.10	31.70	20.55	0.27	0.43	0.35	0.03	0.11	0.06
Rainfed Agricultural Land	23.50	28.10	25.48	0.13	0.28	0.22	0.00	0.02	0.01
Forest	17.80	24.30	22.65	0.24	0.34	0.27	0.00	0.01	0.00

**Table 5:** Amount of soil loss in plot level obtained from RUSLE model.

Watershed	Plot	R-factor	K-factor	LS-factor	C-factor	P-factor	Soil loss (tons ha^−1^ yr^−1^) RUSLE	Soil loss (tons ha^−1^ yr^−1^) Observed	Bias
Tinahukhola	Irrigated	341.95	0.100	2.536	0.21	0.8	14.576	25.7	11.123
	Rainfed	352.8	0.071	4.342	0.21	0.95	21.718	19.4	−2.318
	Forested	279.3	0.085	17.472	0.03	1	12.517	14.3	1.782
Aadhikhola	Irrigated	198.8	0.108	2.536	0.21	0.8	9.152	26.9	17.747
	Rainfed	204.75	0.097	5.060	0.21	0.95	20.054	15.2	−4.854
	Forested	196	0.079	14.517	0.03	1	6.814	6.47	−0.344

Fig. 4 shows the relation between moisture and temperature. The temperature and electrical conductivity change for different land-use classes with the increase in humidity. The increase in moisture content leads to a decrease in temperature for rainfed agricultural land. Electrical conductivity rises, causing the moisture to increase to 0.25 m^3^ m^−3^ before decreasing again. For irrigated agricultural land, electrical conductivity increases linearly with the increase in moisture content, while the relation with temperature is unclear. Both temperature and electrical conductivity increase with the moisture content in forests.

Bulk density provides compactness of the soil, which in turn helps to determine soil disturbance and vulnerability. The bulk densities at 0–15 cm depth show a clear trend in both watersheds (Table 3). The irrigated agricultural land has the highest bulk density, followed by rain-fed plots and forests. In soils at a depth of 15–30 cm, bulk density for all three land-use classes is higher in Aadhikhola than in Tinahukhola.

## 4 Discussion

Among the different soil erosion models available, RUSLE is more widely used than, e.g., USLE and MUSLE because of its versatility and broad usability. RUSLE has been progressively developed and includes an updated research and experimental module to incorporate new data [38]. Improvement in the diagnostic parameters, along with corrections of various inaccuracies, has been made in this model [31,39], resulting in better assessment. For example, there is a correction of the R-factor in the USLE model where if ponded water exists, the model can accurately assess soil cover and management processes, including the influence of topography. The revised model also accounts for soil erodibility due to changes in freeze/thaw and moisture content. In addition, empirical equations have been developed in this model by integrating the updated values of soil conservation practices [49]. Because of these advantages, the application of RUSLE is suitable not only at the local watershed level but also helpful in assessing soil loss at the regional and national levels [22,50].

### 4.1 Estimation of soil loss from the RUSLE model

In the Aadhikhola watershed, we recorded an average rainfall of 2638 mm annually, whereas, in the Tinahukhola watershed, the rainfall was 2010 mm (for 2018). In both watersheds, the average precipitation is higher than the average rainfall in Nepal (1750 mm yr^−1^ [43]). High rainfall contributes to rapid erosion (∼25 tons ha^−1^ yr^−1^) in these watersheds. Additionally, both watersheds are in the hilly region of the mid-range Himalayan mountain chain. The hilly areas with steep slopes aid in the detachment of topsoil, making it easier for downhill transport. This further increases the amount of soil loss in the hilly regions.

In one of the studies using the RUSLE model in the Chitral district, soil erosion has been estimated as high as 78 tons ha^−1^ yr^−1^, and the topographical factor has been identified as the major reason [51]. The authors predict the rate of soil erosion to be worse in the future with changes in land use and climatic conditions. Under RCP (Representative Concentration Pathway) 8.5, it has been predicted that the rate of soil erosion in the same region may reach up to 550 tons ha^−1^ yr^−1^ during 2030–40 [52].

In Nepalese watersheds, variable rates of soil erosion have been documented. A spatial assessment done in the hilly watersheds of Western Nepal reported that the average soil loss in the Aringale watershed in Salyan is 17 tons ha^−1^ yr^−1^ (ranging from 0.03 to 100 tons ha^−1^yr^−1^). The low value is due to less precipitation in the region, contributing to the low R-factor (365–435 MJ mm ha^−1^ h^−1^ yr^−1^) than in Aadhikhola (1071 MJ mm ha^−1^ h^−1^ yr^−1^) and Tinahukhola (733 MJ mm ha^−1^ h^−1^ yr^−1^) watersheds. Similarly, a recent study estimated soil erosion ranging from 0 to 273 tons ha^−1^ yr^−1^, which on average is ∼38.4 tons ha^−1^yr^−1^ for the mid-hills [22]. Consistent with this, the estimates for soil loss in the Aadhikhola watershed in this study are similar. The Gandaki basin recorded an erosion rate of ∼30.7 tons ha^−1^ yr^−1^. Aadhikhola watershed falls within the larger Gandaki Basin. In contrast to Aadhikhola, the Chure/Siwalik region covered by the Tinahukhola watershed indicate a low soil erosion rate (∼6.9 tons ha^−1^ yr^−1^). The Terai floodplains in the Tinahukhola watershed have the lowest erosion rate (∼0.1 tons ha^−1^ yr^−1^). It is suggested that for young mountain chains, the rate of soil erosion up to 25 tons ha^−1^ yr^−1^ is acceptable [53]. Erosion rates in our watersheds are very close to this number or exceeded the proposed limit (>25 tons ha^−1^ yr^−1^), highlighting the vulnerability to soil loss and the immediate need for protective measures that are needed in this region. In particular, most residents in the mid-hills are farmers whose livelihood depends on agriculture; excessive erosion of topsoil from arable land will directly affect their livelihoods.

Using cosmogenic nuclides, an erosion rate equivalent to 1.6 mm/year (average for 15 years) in the Narayani basin was estimated [54]. The authors indicated that this area has a glacial landscape and limited topsoil thickness and supply. Hence, soil erosion contributes ca. 10% of total erosion. However, this study covers large river systems, e.g., Kaligandaki, Trishuli, Marshyangdi, and Narayani, all of which are glacial-fed. The small fluvial watersheds investigated in this study are, however, different. These small watersheds have significant proportions of cultivable land with variable soil depths and gentle slopes. These watersheds are impacted intensely by anthropogenic (land use changes) and natural changes (e.g., erosion and precipitation). Notably, the intense monsoon showers cause more soil erosion than sheet and rill erosion [54]. In addition, landslides during heavy monsoons could contribute to additional soil loss and discharge into these rivers. Although RUSLE underestimates the erosion rate in watersheds with frequent landslides, estimation from this model provides valuable insights. Nonetheless, additional modules must be incorporated into the model to comprehensively assess landslide impacts, mass wasting, and complex erosional processes in heterogenous landscapes [55].

This study divided the watershed into various severity classes based on the soil erosion values (Table 6). The different classes followed the established range [56] and included: very slight (<5 tons ha^−1^ yr^−1^), slight (5–15 tons ha^−1^ yr^−1^), moderate (15–30 tons ha^−1^ yr^−1^) , severe (30–50 tons ha^−1^ yr^−1^), and very severe (>50 tons ha^−1^ yr^−1^) soil loss [57]. Table 6 summarizes the severity of the soil erosion in both of the watersheds in this study. In the Tinahukhola watershed, nearly 50% of the land belongs to the low erosion class according to the soil loss classification scheme [57]. These areas are mainly in the southern part of Nepal, covering the flat Terai plains, and gentle slopes account for low soil erosion. In contrast, nearly 20% of the land cover is affected by severe soil erosion. Such areas are located in the sloping hilly regions in the northern part of the watershed. Thus, in the Tinahukhola watershed, with diverse geomorphological characteristics, experiences a wide range of soil erosion intensity. However, we notice that the Aadhikhola watershed is more vulnerable to soil loss than the Tinahukhola watershed. The Aadhikhola watershed has only 7% of its land belonging to the low soil loss category, while nearly 70% of its land belongs to the class severe and very severe category. This clearly shows the higher vulnerability of soil erosion in the Aadhikhola watershed, implying immediate soil conservation needs in the region.

**Table 6:** Classification of area according to severity class of soil loss.

Soil severity class	Soil loss (tons ha^−1^ yr^−1^)	Tinahukhola watershed		Aadhikhola watershed	
		Area (sq. km.)	Percentage	Area (sq. km.)	Percentage
Very slight	0–5	283.78	45.64	27.4563	6.98
Slight	5–15	52.48	8.44	46.0134	11.71
Moderate	15–30	110.5146	17.77	55.5624	14.14
Severe	30–50	51.55	8.29	129.7791	33.02
Very severe	>50	123.33	19.83	134.1189	34.13

### 4.2 Organic matter and erosion

The organic matter in soil increases infiltration rates of precipitation through soil horizons. The high infiltration rates decrease surface runoff and reduce soil erosion and the K-factor by forming stable soil aggregates with organic matter [58,59]. In general, loss of soil organic matter due to degradation (from land-use practices) can significantly alter its physical properties, namely pore size, bulk density, aggregation, and water-holding capacity in soils [60]. Therefore, we estimated the OC flux for both watersheds [20]. The annual transfer of OC from Tinahukhola is higher than Aadhikhola. However, after normalization concerning their size, Aadhikhola has a higher OC flux (1.67–1.07 ton km^−2^ yr^−1^) than Tinahukhola (1.24–1.05 ton km^−2^ yr^−1^). The erosion rates estimated by RUSLE corroborates trends in suspended sediment load and POC flux [20] in these watersheds. However, the annual suspended sediment load for these watersheds is low compared to the RUSLE values because in those research bedload portion has not been included.

Most of the OC flux is reported from input derived from various terrigenous sources, including old soil. Thus, the K factor in catchments could have variable results based on the parent soil type [58]. They conclude that the K-factor depends mainly on texture in granitic terrains, whereas in limestone-derived soils, OM plays a crucial role. Since both watersheds are carbonate (limestone) dominated sites, OM is most likely the principal factor influencing the K factor when using the RUSLE model for estimating soil erosion.

### 4.3 Change in soil loss and rainfall

In both watersheds, there is clear evidence that rainfall is a significant driver for regulating erosion rates. For example, the Tinahukhola watershed depicts a trend coinciding proportionally between rainfall and erosion rates from 1978 to 2017 (Fig. 5B). A similar trend exists in the Aadhikhola watershed except for a few years when this relationship does not hold (Fig. 5A). For example, the annual precipitation increased from 2468 mm (1978–1982) to 2994 mm (2013–2017) in the Aadhikhola watershed. Similarly, the yearly precipitation increased from 1799 mm (1978–1982) to 2160 mm (2013–2017) in the Tinahukhola watershed. This indicates a steady increase in rainfall in recent years.

Because precipitation is closely correlated with soil loss, this is consistent with the increasing soil loss of 37.7 tons ha^−1^ yr^−1^ (1978–1982) to 43.9 tons ha^−1^yr^−1^ (2013–2017) in the Aadhikhola watershed. Likewise, in the Tinahukhola watershed, soil loss changes from 21.9 tons ha^−1^yr^−1^ (1978–1982) to 25.9 tons ha^−1^ yr^−1^ (2013–2017). Even though the amount of rainfall and rate of soil erosion seems to increase, this is not significant for both Aadhikhola (F_significance_ = 0.56 > α = 0.05) and Tinahukhola (F_significance_ = 0.98 > α = 0.05). In this study, while the amount of precipitation is accounted for, the weather stations did not report its intensity. In this context, we refer to a recent investigation [56], whereby the authors indicate that increased precipitation intensity coincides with a higher soil erosion rate.

### 4.4 Soil loss from erosion plots

Due to the difference in temporal coverage, we observe higher precipitation in Tinahukhola (∼900 mm during 15 observations). In contrast, in Aadhikhola, this is ∼500 mm during 13 observations (Table 4). Even though there is a difference in precipitation in both watersheds, the amount of soil loss is very close. Notably, soil compaction affects soil loss [61], and bulk density in the soil correlates positively with soil loss [62]. In this study, bulk density in the Aadhikhola watershed is higher than in the Tinahukhola watershed for all three land-use activities. Hence, it is likely that differences in the physical property of soil affected the variable soil loss observed in the catchments.

Similarly, various land-use practices impact soil loss in both watersheds. The highest amount of soil loss occurs in the irrigated agricultural plot, followed by the rainfed agricultural plot (Table 4). Forests have the least amount of soil loss. The irrigated agricultural land is highly disturbed due to frequent tillage during the crop cycles. Thus, human activities and land use changes affect soil quality and increase the rate of soil erosion, which is also prevalent in other parts of the mid-Himalayan region in Nepal [63].

Since the plots with different land use are in proximity (within 1 km^2^), the decrease in rainfall recorded in the forests should be due to the interception of precipitation by vegetation cover, specifically its crown. The interception not only reduces the amount of rainfall reaching the ground but also reduces the intensity with which the raindrops hit the ground or, in short, reduces the energy in the raindrop. This, in turn, decreases soil detachment and reduces soil loss.

Even though the RUSLE model is quite reliable at estimating soil loss, it could not depict the local conditions, especially for the irrigated agricultural land in our case. We saw considerable differences between the estimated and observed values. For both watersheds, we found that the observed values were higher than the estimated values for irrigated agricultural land. The main contributing factor to this might be the land use management factor. While the irrigated and rainfed agricultural land has different intensities of land use, both were assigned the exact value of the C-factor, which is 0.21. The irrigated agricultural land faces more intense agricultural practice with 2–3 crops in a year, whereas the rainfed agricultural land has only 1–2 crops in a year. This means frequent the tillage is done in irrigated agricultural land compared to rainfed croplands. Thus, we anticipate that the soil is more fragile in the irrigated agricultural land.

### 4.5 Physical properties of soil and their implication in soil loss

Variable soil characteristics present in different land-use types lead to variations in the rate of soil erosion. It is well known that different land-use practices affect physical conditions in soil [45]. The soils with a higher level of disturbance, i.e., soils from agricultural land, have a low quality (high bulk density). In contrast, soils with minor disturbances have a better quality (low bulk density) [45]. Consistent with this observation, we found similar results in our study. The irrigated agricultural land with a high level of disturbance has the highest bulk density in the topsoil, followed by the rainfed agricultural land. In contrast, forests have the lowest bulk density, signifying less anthropogenic disturbance. Another study documented that physical properties in soils affect soil loss [61]. Soils with high bulk density have high soil erosion. Consistent with this, irrigated agricultural land in our study has the highest rate of soil erosion (Table 4), followed by rainfed agricultural land; the lowest rate of soil erosion occurs in forests. The disturbance and soil bulk density followed the trend: irrigated agricultural land > rainfed agricultural land > and forests.

The irrigated agricultural land has a relatively higher amount of moisture content. The high moisture content in the soil makes it more vulnerable to soil loss. The high moisture content blocks all the pores resulting in water-logged conditions, and preventing the percolation of rainwater. This results in more surface runoff. The high surface runoff, in return, transports more soil from the catchment. In this study, with the increase in moisture content, the temperature declined, whereas the electrical conductivity increased. There has been the practice of using a massive amount of chemical fertilizers to increase the productivity of paddy [64]. This may be the major contributor to the observed high levels of electrical conductivity, i.e., 0.06 mS cm^−1^ (Table 3). Temperature decreases with the increase in moisture content, and less vegetation cover increases the evaporation rate [65]. These factors have led to less moisture content in the rainfed agricultural soils (0.22 m^3^ m^−3^).

Even though forests have low average moisture content and temperature than irrigated agricultural land, the value is higher than that of rainfed agricultural land. The forest soil is found to have a higher water-holding capacity, at least up to a depth of 40 cm [,[[66], [67]]]. Although forest soil has a high moisture content, soil loss is expected to be high due to anthropogenic disturbances in the rainfed agricultural plots. In both watersheds, the sloping area covered by forests is more than 60% [20]. Even with most of the area under forest cover in both watersheds, soil loss exceeds the acceptable limits. Moreover, urbanization trends in Nepal indicate that forests are rapidly converted into built-up areas and/or agricultural croplands. Thus, land use further increases the vulnerability to soil loss in these mountainous terrains. As both of our watersheds are vulnerable to extreme rainfall events, steep slopes, and landslides, the changeover in land use further increases topsoil erosion in these watersheds.

## 5 Conclusions

In this study, we identified areas with a high vulnerability to soil erosion in the mid-hills of Nepal. We analyzed the soil loss from two small representative watersheds using the RUSLE model and experimental soil erosion plots. The experimental field plots were set up in areas with three different land-use activities, i.e., irrigated agricultural land, rainfed agricultural land, and forests in both watersheds. Soil loss is higher in the Aadhikhola watershed than in the Tinahukhola watershed. The primary reason is the high precipitation in Aadhikhola and the steep mountain slopes. Based on land use, irrigated agricultural land had the highest rate of soil erosion, followed by rainfed agricultural land. The forests with high vegetation cover have the least amount of soil loss. The soil loss correlated positively with compactness in the soil. Soils with higher bulk densities are more prone to soil loss. We tested whether the amount of soil loss has changed due to the change in rainfall patterns over the last 40 years, but the results are statistically insignificant. Finally, while the RUSLE model can estimate soil loss with less bias for forests and rainfed agricultural land, the model tends to underestimate soil loss from irrigated agricultural land. Thus, we recommend revising the model that captures the local conditions and farming practices in regions like Nepal. This would allow relevant stakeholders and policymakers to plan for better soil conservation strategies in erosion-prone areas in the mid-hills of Nepal. For examples, a plantation in a barren land, reduced tillage practice, terrace farming in sloping lands, etc. are recommended as simple yet most effective measures for reducing soil erosion.

## Declaration of competing interest

The authors declare that they have no known competing financial interests or personal relationships that could have appeared to influence the work reported in this paper.

## References

[1] Brady N.C., Weil R.R., Weil R.R. (2008).

[2] Lal R. (2001). Soil degradation by erosion. Land Degrad. Dev..

[3] Panagos P., Borrelli P., Meusburger K., Alewell C., Lugato E., Montanarella L. (2015). Land Use Policy Estimating the soil erosion cover-management factor at the European scale. Land Use Pol..

[4] Dregne H.E. (1992). Erosion and soil productivity in asia. J. Soil Water Conserv..

[5] Pimentel D., Burgess M. (2013).

[6] Pimentel D., Harvey C., Resosudarmo P., Sinclair K., Kurz D., McNair M., Crist S., Shpritz L., Fitton L., Saffouri R. (1995). Environmental and economic costs of soil erosion and conservation benefits. Science.

[7] Li P., Mu X., Holden J., Wu Y., Irvine B., Wang F., Gao P., Zhao G., Sun W. (2017). Comparison of soil erosion models used to study the Chinese Loess Plateau. Earth Sci. Rev..

[8] Joshi J., Bhattarai T.N., Sthapit K.M., Omura H. (1998). Soil erosion and sediment disaster in Nepal--a review. J. Fac. Agric. Kyushu Univ..

[9] Maximillian J., Brusseau M.L., Glenn E.P., Matthias A.D. (2019).

[10] Sudmeier-Rieux K., McAdoo B.G., Devkota S., Rajbhandari P.C.L., Howell J., Sharma S. (2019). Invited perspectives: mountain roads in Nepal at a new crossroads. Nat. Hazards Earth Syst. Sci..

[11] Dabral P.P., Baithuri N., Pandey A. (2008).

[12] MoFE (2019). http://mofe.gov.np/downloadfile/MOFE_2019_ClimatechangescenariosforNepal_NAP_1562647620.pdf.

[13] Chalise D., Kumar L., Kristiansen P. (2019). Land degradation by soil erosion in Nepal: a review. Soil Syst.

[14] Timilsina R.H., Ojha G.P., Nepali P.B., Tiwari U. (2019). Agriculture land use in Nepal: prospects and impacts on food security. J. Agric. For. Univ..

[15] Atreya K., Sharma S., Bajracharya R.M., Rajbhandari N.P. (2006). Applications of reduced tillage in hills of central Nepal. Soil Tillage Res..

[16] Wischmeier W.H., Smith D. (1978).

[17] Chalise D., Kumar L., Shriwastav C.P., Lamichhane S. (2018). Spatial assessment of soil erosion in a hilly watershed of Western Nepal Spatial assessment of soil erosion in a hilly watershed of Western Nepal. Environ. Earth Sci..

[18] Bin Ashoor B., Giwa A., Hasan S.W. (2018).

[19] Mou Jinze (1981). Eros. Sediment Transp. Meas. Proc. Florence Symp. June 1981, (International Assoc. Hydrol. Sci. IAHS-AISH Publ..

[20] Bhandari R., Routh J., Joshi P., Chhetri S., Joshi R. (2022). Bulk carbon and lignin fingerprinting of catchment sediments transported by mountain rivers in Nepal Himalayas. Catena.

[21] Madden L.V., Wilson L.L., Ntahimpera N. (1998). Calibration and evaluation of an electronic sensor for rainfall kinetic energy. Phytopathology.

[22] Koirala P., Thakuri S., Joshi S., Chauhan R. (2019). Estimation of soil erosion in Nepal using a RUSLE modeling and geospatial tool. Geosciences.

[23] Morgan R.P.C. (1985). Soil erosion measurement and soil conservation research in cultivated areas of the UK. Geogr. J..

[24] Bhandari R., Routh J., Sharma S., Joshi R. (2021). Contrasting lipid biomarkers in mountain rivers in the Nepal Himalayas: organic matter characteristics and contribution to the fluvial carbon pool. Geosci. Front..

[25] Kharel L.N. (2002).

[26] Kayastha P., Dhital M.R., De Smedt F. (2012). Landslide susceptibility mapping using the weight of evidence method in the Tinau watershed, Nepal. Nat. Hazards.

[27] Vaidya S.N., Sherchan D.P., Tiwari K.R., Subedi S., Karki K.B., Panday D., Ojha R.B. (2021). Soil types, soil classification, and mapping. The Soils of Nepal.

[28] Dahal R.K. (2006).

[29] Chidi C.L. (2014). Geomorphic determinanats of landuse intensity. Int. Arch. Photogramm. Remote Sens. Spat. Inf. Sci. - ISPRS Arch. XL–.

[30] Chidi C.L., Sulzer W., Pradhan P.K. (2019). Landscape dynamics in the northeast part of Andhikhola watershed, Middle hills of Nepal. Geogr. J. Nepal..

[31] Renard K.G. (1997).

[32] Risal A., Bhattarai R., Kum D., Park Y.S., Yang J.E., Lim K.J. (2016). Application of web ERosivity module (WERM) for estimation of annual and monthly R factor in korea. Catena.

[33] Ganasri B.P., Ramesh H. (2016). Assessment of soil erosion by RUSLE model using remote sensing and GIS-A case study of Nethravathi Basin. Geosci. Front..

[34] Bouyoucos G.J. (1962). Hydrometer method improved for making particle size analyses of soils 1. Agron. J..

[35] Nelson D.W., Sommers L.E. (1996). Total carbon, organic carbon, and organic matter. Methods Soil Anal. Part 3 Chem. Methods..

[36] Williams J.R., Sharply A.N., Cacalator E.-E.P.I. (1990).

[37] Panagos P., Meusburger K., Alewell C., Montanarella L. (2012). Soil erodibility estimation using LUCAS point survey data of Europe. Environ. Model. Software.

[38] Djoukbala O., Hasbaia M., Benselama O., Mazour M. (2019). Comparison of the erosion prediction models from USLE, MUSLE and RUSLE in a Mediterranean watershed, case of Wadi Gazouana (N-W of Algeria). Model. Earth Syst. Environ..

[39] Renard B.K.G., Foster G.R., Weesies G.A., Porter J.I. (1991). Revised universal soil loss equation (rusle). J. Soil Water Conserv..

[40] Wischmeier W.H., Smith D.D. (1978).

[41] Chalise D., Kumar L., Shriwastav C.P., Lamichhane S. (2018). Spatial assessment of soil erosion in a hilly watershed of Western Nepal. Environ. Earth Sci..

[42] Shin G.J. (1999).

[43] Müller M.F., Thompson S.E. (2013). Bias adjustment of satellite rainfall data through stochastic modeling: methods development and application to Nepal. Adv. Water Resour..

[44] Badu M., Ghimire C.P., Bruijnzeel L.A., Nuberg I., Meyer W.S. (2022). Net precipitation, infiltration and overland flow production in three types of community-managed forest in the Mid-hills of East Central Nepal. Trees, For. People..

[45] Begum F., Bajracharya R.M., Sharma S., Situala B.K. (2009). Int. Conf. Emerg. Technol. Environ. Sci. Eng.

[46] Dabral P.P., Baithuri N., Pandey A. (2008). Soil erosion assessment in a hilly catchment of North Eastern India using USLE, GIS and remote sensing, Water Resour. OR Manag..

[47] Martínez-Murillo J.F., Remond R., Ruiz-Sinoga J.D. (2020). Validation of RUSLE K factor using aggregate stability in contrasted mediterranean eco-geomorphological landscapes (southern Spain). Environ. Res..

[48] Rwanga S.S., Ndambuki J.M. (2017). Accuracy assessment of land use/land cover classification using remote sensing and GIS. Int. J. Geosci..

[49] Benavidez R., Bethanna J., Deborah M., Kevin N. (2018). A-review-of-the-Revised-Universal-Soil-Loss-Equation-RUSLE-With-a-view-to-increasing-its-global-applicability-and-improving-soil-loss-estimates. Hydrol. Earth Syst. Sci..

[50] Chen T., qing Niu R., xiang Li P., pei Zhang L., Du B. (2011). Regional soil erosion risk mapping using RUSLE, GIS, and remote sensing: a case study in Miyun Watershed, North China. Environ. Earth Sci..

[51] Maqsoom A., Aslam B., Hassan U., Kazmi Z.A., Sodangi M., Tufail R.F., Farooq D. (2020). Geospatial assessment of soil erosion intensity and sediment yield using the revised universal soil loss equation (RUSLE) model. ISPRS Int. J. Geo-Inf..

[52] Aslam B., Khalil U., Saleem M., Maqsoom A., Khan E. (2021). Effect of multiple climate change scenarios and predicted land-cover on soil erosion: a way forward for the better land management. Environ. Monit. Assess..

[53] Morgan R.P.C. (1986).

[54] Morin G.P., Lavé J., France-Lanord C., Rigaudier T., Gajurel A.P., Sinha R. (2018). Annual sediment transport dynamics in the Narayani basin, Central Nepal: assessing the impacts of erosion processes in the annual sediment budget. J. Geophys. Res. Earth Surf..

[55] Alewell C., Borrelli P., Meusburger K., Panagos P. (2019). Using the USLE: chances, challenges and limitations of soil erosion modelling. Int. Soil Water Conserv. Res..

[56] Chalise D., Kumar L., Kristiansen P. (2019). Land degradation by soil erosion in Nepal: a review. Soil Syst.

[57] Romshoo S.A., Yousuf A., Altaf S., Amin M. (2021). Evaluation of various DEMs for quantifying soil erosion under changing land use and land cover in the Himalaya. Front. Earth Sci..

[58] Liu M., Han G., Li X., Zhang S., Zhou W., Zhang Q. (2020). Effects of soil properties on K factor in the granite and limestone regions of China. Int. J. Environ. Res. Publ. Health.

[59] Al Rammahi A.H.J., Khassaf S.I. (2018). Estimation of soil erodibility factor in rusle equation for euphrates river watershed using GIS. Int. J. GEOMATE..

[60] Ara B., Fak Z., Bilimi T., Besleme B. (2014). The effects of forestation on RUSLE-K factor in lowland ecosystem of semi arid areas in Turkey türkiye ’ deki yarı kurak ova ekosistemlerinde ağaçlandırmanın , yenilenmiş üniversal toprak kayıpları eşitliğindeki toprak erozyon faktörü (K) üzerine. Etkisi.

[61] Liu Y., Fu B., Lü Y., Wang Z., Gao G. (2012). Hydrological responses and soil erosion potential of abandoned cropland in the Loess Plateau, China. Geomorphology.

[62] Feng Q., Zhao W., Wang J., Zhang X., Zhao M., Zhong L., Liu Y., Fang X. (2016). Effects of different land-use types on soil erosion under natural rainfall in the loess plateau, China. Pedosphere.

[63] Tiwari K.R., Sitaula B.K., Bajracharya R.M., Borresen T. (2009). Runoff and soil loss responses to rainfall, land use, terracing and management practices in the Middle Mountains of Nepal. Acta Agric. Scand. Sect. B Soil Plant Sci.

[64] Sampanpanish P., Alam L., Mohamed C.A., Bin Mokhtar M., Han E.C., Huang Y.-C., Lin J.-M., Lin H.-J., Wu J.-Y., Lee C.-C. (2012). others, Use of organic fertilizer on paddy fields to reduce greenhouse gases. Sci. Asia.

[65] Choudhury B.J., Ahmed N.U., Idso S.B., Reginato R.J., Daughtry C.S.T. (1994). Relations between evaporation coefficients and vegetation indices studied by model simulations. Remote Sens. Environ..

[66] Craib I.J. (1929).

[67] Blažka P., Fischer Z. (2014). Moisture, water holding, drying and wetting in forest soils. Open J. Soil Sci..

