# Supplementary material for: Himalayan watersheds in Nepal record high soil erosion rates estimated using the RUSLE model and experimental erosion plots
Source: Heliyon. 2023 May 2;9(5):e15800. doi: 10.1016/j.heliyon.2023.e15800 (PMC10192770; doi:10.1016/j.heliyon.2023.e15800)

| Aadhikhola watershed | | | | | | |
| --- | --- | --- | --- | --- | --- | --- |
|  | Forest | Agricultural | Barren | Built Up | Water Body | Total (User) |
| Forest | 59 | 8 | 3 | 1 | 0 | 71 |
| Agricultural | 5 | 65 | 4 | 0 | 0 | 74 |
| Barren | 2 | 6 | 47 | 2 | 0 | 57 |
| Built Up | 1 | 2 | 4 | 44 | 1 | 52 |
| Water Body | 1 | 0 | 2 | 0 | 46 | 49 |
| Total (Producer) | 68 | 81 | 60 | 47 | 47 | 303 |
|  | | | | | | |
| Overall Accuracy | | | 86.13% | | | |
|  | | | | | | |
|  | | User Accuracy | | | Producer Accuracy | |
| Forest | | 83.09% | | | 86.76% | |
| Agricultural | | 87.83% | | | 80.24% | |
| Barren | | 82.45% | | | 78.33% | |
| Built Up | | 84.61% | | | 93.61% | |
| Water Body | | 93.87% | | | 97.87% | |
|  | | | | | | |
| Row*Column | | | 18989 | | | |
| Total Correct*Total | | | 79083 | | | |
| Total Square | | | 91809 | | | |
|  | | | | | | |
| Kappa Coefficient | | | 0.825 | | | |

# Supplementary Tables

Supplementary Table 1- Accuracy assessment of Aadhikhola LULC

Supplementary Table 2- Accuracy assessment of Tinahukhola LULC

| Tinahukhola watershed | | | | | | |
| --- | --- | --- | --- | --- | --- | --- |
|  | Forest | Agricultural | Barren | Built Up | Water Body | Total (User) |
| Forest | 57 | 5 | 2 | 1 | 1 | 66 |
| Agricultural | 4 | 53 | 2 | 0 | 1 | 60 |
| Barren | 1 | 2 | 45 | 7 | 0 | 55 |
| Built Up | 1 | 3 | 4 | 42 | 0 | 50 |
| Water Body | 0 | 0 | 0 | 1 | 46 | 47 |
| Total (Producer) | 63 | 63 | 53 | 51 | 48 | 278 |
|  | | | | | | |
| Overall Accuracy | | | 87.41% | | | |
|  | | | | | | |
|  | | User Accuracy | | | Producer Accuracy | |
| Forest | | 86.36% | | | 90.47% | |
| Agricultural | | 88.33% | | | 84.12% | |
| Barren | | 81.81% | | | 84.90% | |
| Built Up | | 84% | | | 82.35% | |
| Water Body | | 97.87% | | | 95.83% | |
|  | | | | | | |
| Row*Column | | | 15659 | | | |
| Total Correct*Total | | | 67554 | | | |
| Total Square | | | 77284 | | | |
|  | | | | | | |
| Kappa Coefficient | | | 0.842 | | | |

Supplementary Table 3 - Details of the DHM stations used for precipitation data

| Name of Station | Watershed | Station type | Latitude | Longitude | Elevation (masl) |
| --- | --- | --- | --- | --- | --- |
| Butwal | Tinahukhola | Climate | 27.86366**°** | 83.53871**°** | 1183 |
| Dandaswara |  | Climate | 27.69439**°** | 83.46631**°** | 180 |
| Hattilung |  | Precipitation | 27.8075**°** | 83.31314**°** | 1640 |
| Karki Neta |  | Precipitation | 27.72169**°** | 83.62022**°** | 1127 |
| Baldyangadi | Aadhikhola | Precipitation | 28.17727**°** | 83.7482**°** | 1642 |
| Panchamul |  | Climate | 28.09874**°** | 83.87251**°** | 871 |
| Panchase |  | Climate | 28.08492**°** | 83.92201**°** | 1316 |
| Syangja |  | Precipitation | 28.13558**°** | 83.7702**°** | 1575 |
| Tansen |  | Climate | 28.22897**°** | 83.79723**°** | 2492 |

Supplementary Table 4- Description of measurement by EM-50 sensors for f temperature, moisture, and electrical conductivity

| Land use | Data available from | Data available to |
| --- | --- | --- |
| Irrigated Agricultural land | June 10, 2018 | May 18, 2019 |
| Rainfed Agricultural land | April 30, 2019 | August 4, 2019 |
| Forested Land | April 30, 2019 | August 4, 2019 |

# Supplementary Figures

Figure 1: Rainfall erosivity map (model output); A – Aadhikhola watershed, B – Tinahukhola

watershed


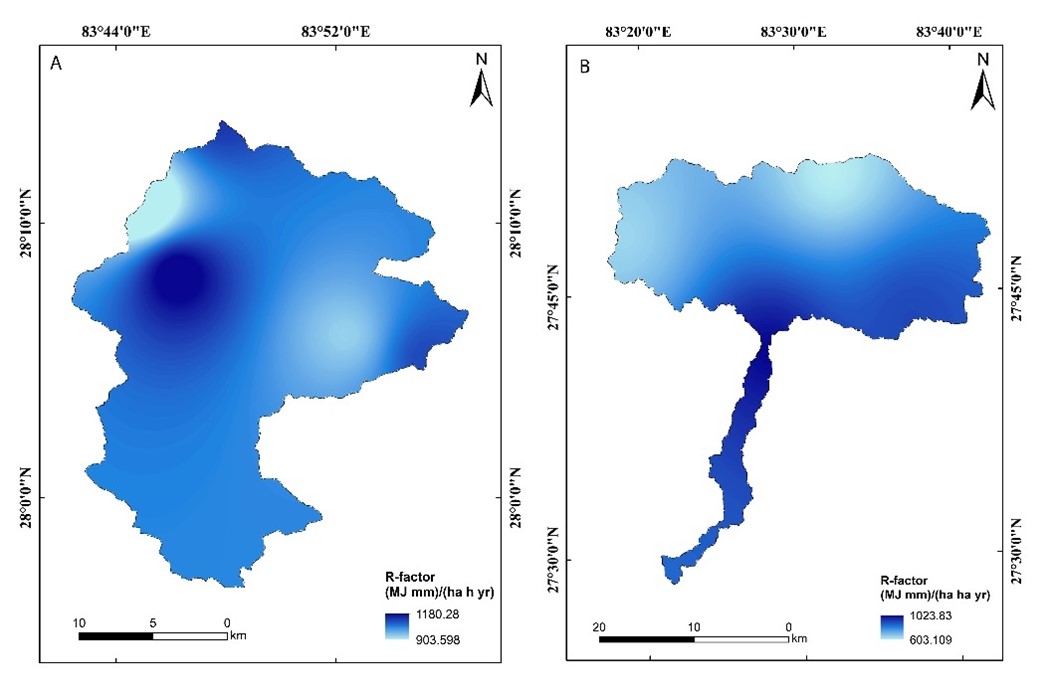


Figure 2: Soil erodibility factor map (model output): A – Aadhikhola watershed, B – Tinahukhola watershed


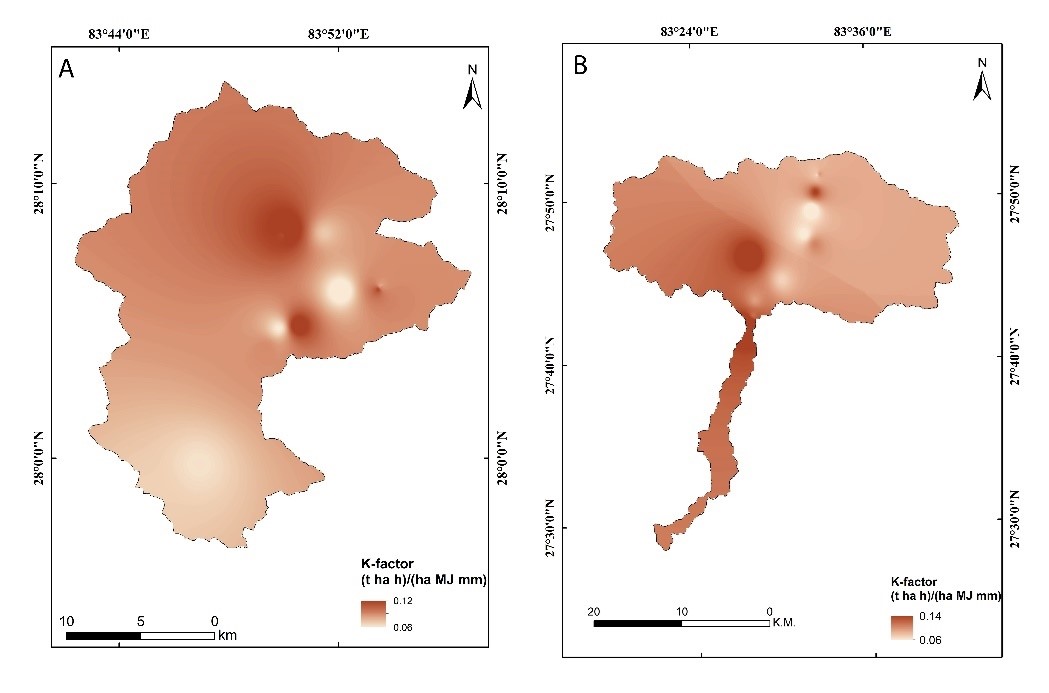


Figure 3: Slope length and Steepness factor map (model output): A – Aadhikhola watershed, B – Tinahukhola watershed


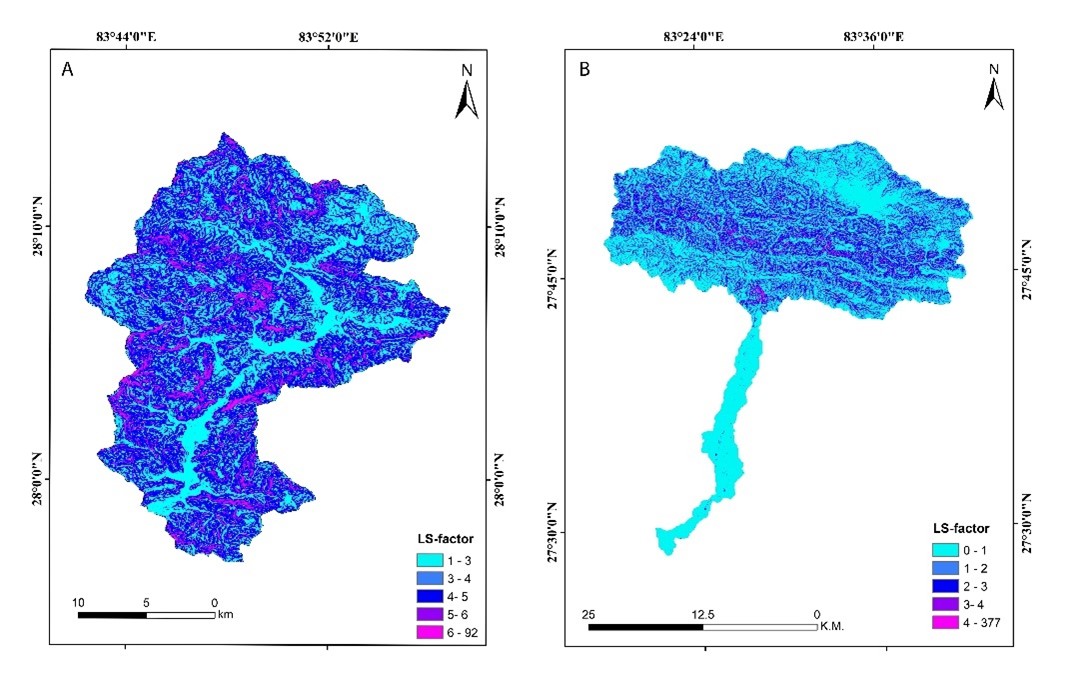


Figure 4: Cover management factor map (model output): A – Aadhikhola watershed, B – Tinahukhola watershed


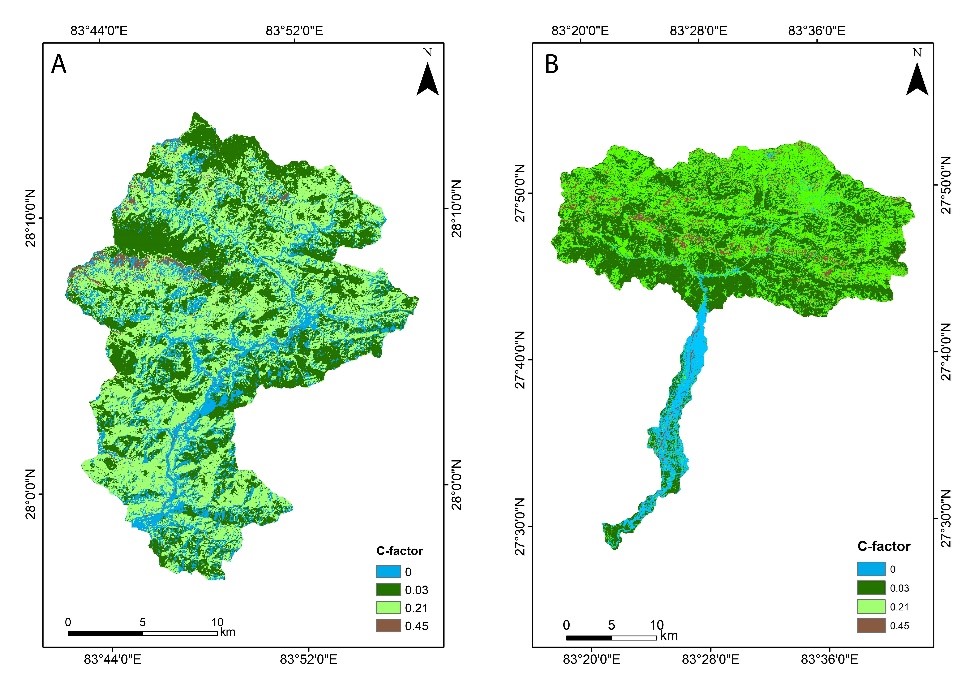


Figure 5: Support practice factor map (model output): A – Aadhikhola watershed, B – Tinahukhola watershed


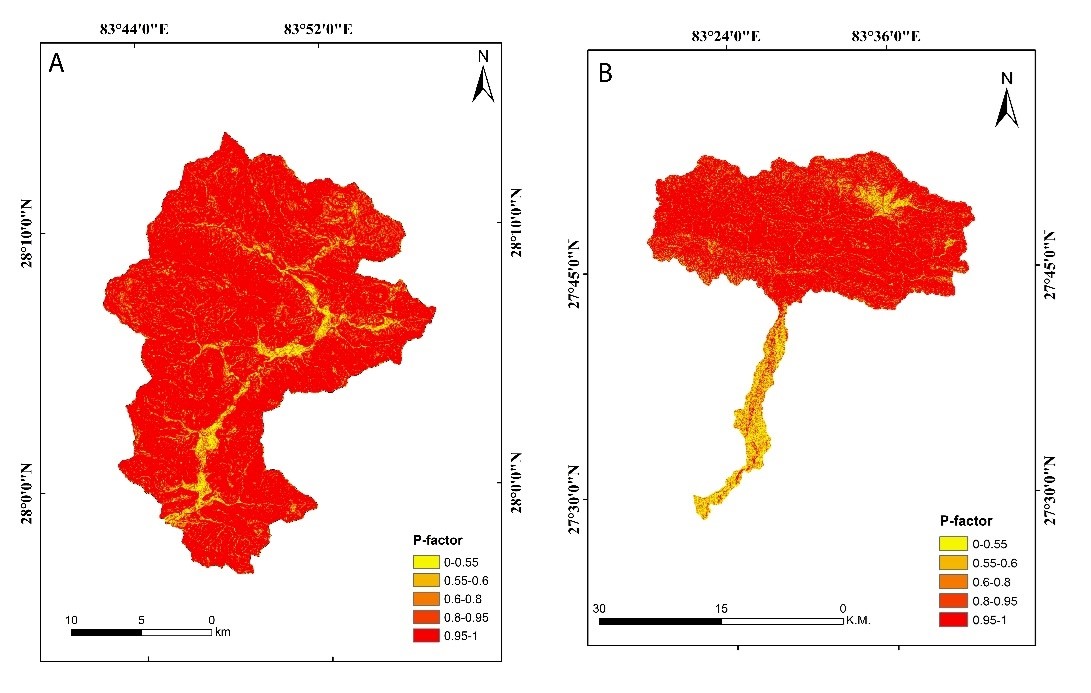

Supplement: Supplementary Files [file mmc1.docx]
